# Sorting protein decoys by machine-learning-to-rank

**DOI:** 10.1038/srep31571

**Published:** 2016-08-17

**Authors:** Xiaoyang Jing, Kai Wang, Ruqian Lu, Qiwen Dong

**Affiliations:** 1 School of Computer Science, Fudan University, Shanghai 200433, People’s Republic of China; 2 College of Animal Science and Technology, Jilin Agricultural University, Changchun 130118, People’s Republic of China; 3 Institute for Data Science and Engineering, East China Normal University, Shanghai 200062, People’s Republic of China

## Abstract

Much progress has been made in Protein structure prediction during the last few decades. As the predicted models can span a broad range of accuracy spectrum, the accuracy of quality estimation becomes one of the key elements of successful protein structure prediction. Over the past years, a number of methods have been developed to address this issue, and these methods could be roughly divided into three categories: the single-model methods, clustering-based methods and quasi single-model methods. In this study, we develop a single-model method MQAPRank based on the learning-to-rank algorithm firstly, and then implement a quasi single-model method Quasi-MQAPRank. The proposed methods are benchmarked on the 3DRobot and CASP11 dataset. The five-fold cross-validation on the 3DRobot dataset shows the proposed single model method outperforms other methods whose outputs are taken as features of the proposed method, and the quasi single-model method can further enhance the performance. On the CASP11 dataset, the proposed methods also perform well compared with other leading methods in corresponding categories. In particular, the Quasi-MQAPRank method achieves a considerable performance on the CASP11 Best150 dataset.

In the last two decades, with the increasing number of experimental structures and the promotion of computational techniques and computing power, various protein three-dimensional structure prediction methods have been developed and a lot of progress has been made in this area^1^. It is possible to generate numerous predicted models for a given protein sequence, and these models often span a broad range of accuracy spectrum and need to be annotated with accurate quality estimation for specific biomedical applications^2^. However, ranking the predicted models correctly and selecting the best predicted model from the candidate pool remain as challenging tasks.

With the rapid increase in computing power, a greater number of models for a certain sequence can be predicted, consequently, evaluating the quality of a certain protein model in perspective is emerging as a problem that cannot be ignored. Since CASP7 (7th Community Wide Experiment on the Critical Assessment of Techniques for Protein Structure Prediction)^3^, a new prediction category evaluating the quality of predicted protein models and the reliability of predicting certain residues in the structure was implemented, which is called model quality assessment (MQA). Over the past years, a number of methods have been developed to address this issue^4^,^5^, and these methods could roughly be divided into three categories: single-model methods, clustering-based (or consensus-based) methods and quasi single-model methods. Single-model methods evaluate the model quality using the inputted model only^6^,^7^,^8^. Three conceptual approaches are often used in this category: the physical model, the statistical model and the comparison between the predicted properties and the actual properties extracted from the model, such as the uniformity of secondary structures, the solvent accessibility, the contact map, residue environment and other features. Many machine learning algorithms have been used to get an accurate estimation of the model quality from various features, such as support vector machine^9^, deep learning^10^, random forest^11^ etc. The basic assumption of the clustering-based methods is that the near-native conformations have more chance to be re-sampled by structure prediction methods than the low quality structure models, so the conformations predicted with high frequency are more likely to be correct than structural patterns occurring in only a few models. The clustering-based methods often use clustering algorithm to cluster a set of decoys generated by structure prediction programs for a certain target sequence^12^,^13^,^14^. Previous studies have found that clustering-based methods generally outperform single-model methods when numerous models are available from several different structure prediction methods^15^,^16^, which is also confirmed by the CASP (Critical Assessment of protein Structure Prediction)^4^,^5^,^17^. However, because of the strong dependence of model quality, the clustering-based methods will perform poorly and fail to select the best model from the decoy set if most of the models are of low qualities (differing much from the native structure). The quasi single-model methods adopt the single-model methods’ strategy to identify a few high-quality models as references or predict some models as references, and then evaluate the subsequent models by comparing them with the reference models^18^,^19^. In some sense, the quasi single-model methods take into account various features and occurrence frequency of the model at the same time, and can be seen a combination of the single-model methods and the clustering-based methods. There are also some specific methods for evaluating model quality of special proteins, such as trans-membrane proteins^20^,^21^. A lot of servers which usually integrate multiple assessment strategies have been developed for the model quality assessment of proteins^22^,^23^,^24^.

For a target protein, the problem of decoy model quality assessment could be deemed as ranking the decoy models based on their similarities to the corresponding native structure. These similarities can be measured by various structural alignment scoring methods, such as GDT_TS score (global distance test total score)^1^, TM-score^25^, Max-sub score^26^, LGA score^27^ etc. A similar problem has been extensively studied in information retrieval^28^. The goal of information retrieval is to rank every relevant document to reflect the relevance or the importance of the document with the specific query. In recent years, the learning-to-rank methods dealing with such task have achieved great performance^29^ and have been successfully applied to relevant fields such as document retrieval^30^, collaborative filtering^31^, spam detection^32^, etc. The learning-to-rank methods combine information retrieval techniques with machine learning theory, and their goal is to obtain a ranking strategy from the training set using various algorithms and rank documents in the test set. In view of its good performance, learning-to-rank methods have been applied in many bioinformatics tasks including disease name normalization^33^, biomedical document retrieval^34^, gene summary extraction^35^, protein folding energy designing^36^, etc.

In this study, a novel method based on learning-to-rank has been developed for protein model quality assessment, which sorts the decoy models to indicate the relative qualities for a target protein. The preliminary test on a well-developed decoy model dataset shows that the proposed method consistently outperforms other methods whose outputs are taken as features. The performance are further improved by using the quasi single-model strategy. Further test on the CASP11 (11th Community Wide Experiment on the Critical Assessment of Techniques for Protein Structure Prediction) dataset shows that, this quasi single-model method based on learning-to-rank could get better results in comparison with the top established methods. The following paper is organized as follows: Section 2 presents the results and discussions, Section 3 describes the methodology and the dataset, and the conclusion and further directions are shown in Section 4.

## Results and Discussions

### Overview of the proposed methods

**Figure 1:**
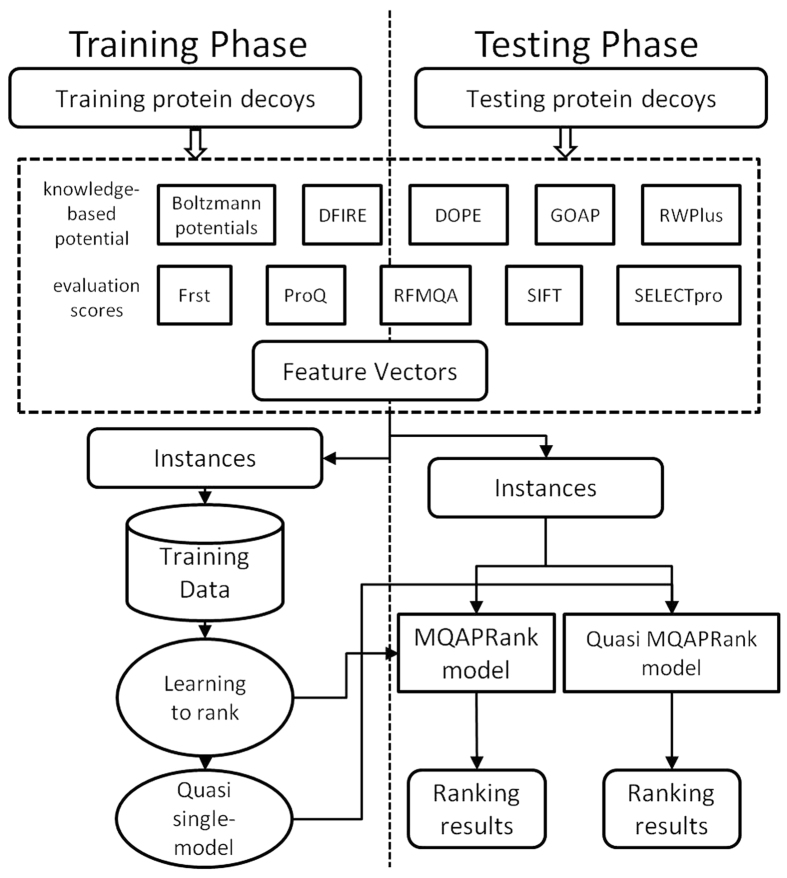
The overall flowchart of the proposed methods.

The model quality assessment of protein decoys is formulated as a ranking problem, as the protein decoy models are sorted by their similarities with the corresponding native structure. Such similarities can be measured by various structure comparison programs. The GDT_TS score^1^ and the TM-Score^25^ are adopted in this study, and their differences are also compared. A variant of features are first extracted from the decoy models, including the knowledge-based potentials and the evaluation scores of other model quality assessment programs. Each decoy is represented as a feature vector in which the elements are taken from the features. The training or test instances are pairs of decoys from the same proteins. These instances are then inputted into learning to rank algorithm to construct the learning to rank model which is subsequently used to predict the relative sorting relation of any two decoys from the same protein. The proposed method is named as MQAPRank for short. A quasi single-model method is also implemented, which takes the first five models ranked by the MQAPRank method as the reference models and the predicted qualities of other models are the average GDT_TS score (or TM-score) of the target models with the reference models. The corresponding method is named as Quasi-MQAPRank with prefix “quasi”. The overall flowchart of the proposed methods are illustrated in Fig. 1.

### Evaluation metrics

The majority of the evaluation metrics and assessment procedures that we used are the same as those used in the official assessment of previous and recent CASP^4^,^5^,^37^. These include:

1. The PMCC (Pearson’s product–moment correlation coefficient) of the predicted model quality (the reciprocal of the rank) with the observed GDT_TS score (or TM-score) values on a decoy set.

As mentioned in CASP11^5^, predicted and observed data can be compared on a target-by-target basis or by pooling models from all targets together, and the two assessment approaches show different performance of the methods. Consequently, we used both of them and named them as wmPMCC (weighted mean of PMCC) and PMCC respectively.

The wmPMCC is calculated following the way in the CASP7^38^. Firstly, the PMCC is transformed into an additive quantity applying the Fisher’s transformation (equation (1)):


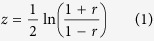


where r is the PMCC; z is the normally distributed variable transformed from r, and the standard error of z is equation (2):


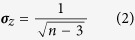


where n is the sample size.

Then, we could get the wmPMCC by using the inverse formula (equation (3)):


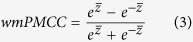


where 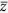 denotes the arithmetic mean score of a given set of z values.

2. The ROC (Receiver Operating Characteristic) analysis of the ability to discriminate between good and bad models, a model is defined as a good model if the observed GDT_TS score is larger than 50 (TM-score larger than 0.5), otherwise it is defined as a bad model. The AUC (area under the curve) of each ROC curve is also calculated.

3. Loss in quality between the best available and the estimated best model, related to the capability of selecting the very best model in a decoy set (Loss).

4. The ranking of the best model in the model list for each target protein is counted and the total number of ranking in the first place is named as Top.

### Performance comparison on 3DRobot dataset

In order to verify the ability of our methods for model quality assessment, a preliminary benchmark evaluation of the proposed methods is performed on the 3DRobot dataset (200 target proteins) by using five-fold cross-validation. The five-fold cross-validation is done as follows: Firstly, 200 protein targets are divided into five parts randomly, and each part contains 40 protein targets. Then, we select one part (the decoy models of 40 targets) as a test set and the remaining four parts (the decoy models of 160 targets) as a train set. This process repeats five times, and each part has been used as a test set. Finally, prediction results of five test parts are integrated together, and assessment was made on it.

**Figure 2:**
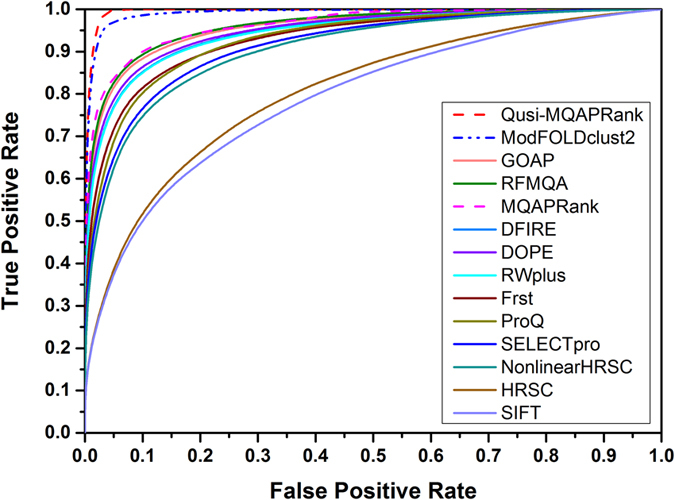
The ROC curves of compared methods on the 3DRobot dataset based on GDT_TS score. The ModFOLDclust2 is a clustering method, other compared methods are listed in “feature extraction” section.

Our method sorts the decoy models by their qualities, which means the model will get a higher score if it’s more similar to the native structure of the target protein. Therefore the inverse of the score can be used to indicate the relative sorting index of each model. We compared our method with previous well-developed methods based on two different structure similarity metrics: GDT_TS score (global distance test total score)^1^ and TM-score^25^. The evaluation results are shown in Table 1, which is based on the GDT_TS score (the corresponding results based on TM-score are shown in Supplementary Table S1), and the ROC curves based on GDT_TS score are provided in Fig. 2 (the corresponding ROC curves based on TM-score are provided in Supplementary Fig. S1). It should be noted that potentials are negatively correlated with the model quality, so the absolute value of PMCC is used here.

It clearly shows that our method consistently outperforms all other methods whose outputs are taken as features in terms of almost all evaluation metrics (a little bit of poor in the Top metric). Two factors contributed to the success of the proposed method. One is the learning-to-rank framework that can give the relative quality of a set of decoy models. The other is the features which are the complementary outputs of various methods.

In previous studies, many results show that the cluster-based methods outperform the single-model methods when numerous models are available. The basic assumption of the clustering-based methods is that the conformations are more likely to be correct when more frequently predicted, so the best model should be in the largest cluster and be the nearest point of cluster center. We benchmark a clustering method ModFOLDclust2^39^ on the 3DRobot dataset. According to the results, the ModFOLDclust2 outperforms other single model methods on correlation coefficient and ROC metrics. But on Loss and Top metrics, it performs poorly, especially in selecting the best decoy model. Here, we extend the single-model method by using the top five models as the reference models. The qualities of other models are calculated by the average similarity with these reference models based on the corresponding structure similarity metric. The quasi single-model method is named with prefix “quasi”. As shown in Table 1 (and Supplementary Table S1) and Fig. 2 (and Supplementary Fig. S1), the quasi single-model method can further improve the performance in comparison with the single-model method, and achieve near 100% PMCC and AUC values.

### Performance comparison on CASP11 dataset

With reference to the rules of CASP^5^, we use CASP10 (10th Community Wide Experiment on the Critical Assessment of Techniques for Protein Structure Prediction) dataset as the training set of our methods and make tests on the CASP11 dataset (Best150 dataset and Select20 dataset). Referring to the assessment results of CASP11^5^, we select the state-of-the-art groups as references according to their wmPMCC on the Best150 dataset in different method categories. We get fifteen reference methods, six methods (Pcons-net, Wallner, DAVIS-QAconsensus, MULTICOM-REFINE, ModFOLDclust2 and MQAPmulti) are the clustering-based methods, five methods (MQAPsingleA, MQAPsingle, ModFOLD5_single, nns and ConsMQAPsingle) are the quasi single-model methods, and four methods (MULTICOM-CLUSTER, VoroMQA, MULTICOM-NOVEL and ProQ2) are the single-model methods.

**Figure 3:**
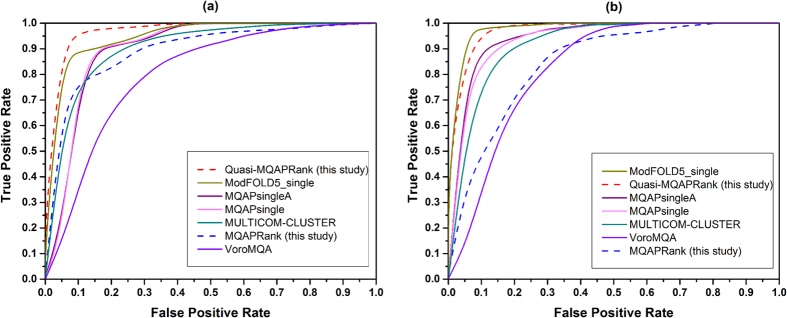
The ROC curves of compared methods on the CASP11 dataset based on GDT_TS score. (**a**) The ROC curves for Best150 dataset and (**b**) the corresponding AUCs for Select20 dataset.

Table 2 shows the performance of our methods and the reference methods on the CASP11 dataset (Best150 dataset and Select20 dataset) based on GDT_TS score (the results based on TM-score are shown in Supplementary Table S2). The ROC curves of four state-of-the-art methods in corresponding categories and the two proposed methods based on GDT_TS score are provided in Fig. 3 (the ROC curves based on TM-score are shown in Supplementary Fig. S2). In general, when numerous models are available, the clustering-based methods outperform single-model and quasi single-model methods in previous studies, this is also confirmed in Table 2 (also in Supplementary Table S2) that the clustering-based methods perform well based on most evaluation metrics on the Best 150 dataset. But the proposed quasi single-model method Quasi-MQAPRank has a comparative and even better performance, especially on wmPMCC and Loss metrics. In Supplementary Table S2 (TM-score), there is a similar result and the Quasi-MQAPRank method shows a particularly better performance on the wmPMCC and Loss metrics. In CASP11, the ProQ2 method is the best single method on the Loss metric (6.34) on the Best 150 dataset, but the proposed single method MQAPRank shows a better performance (4.46). On the Select20 dataset, the proposed method Quasi-MQAPRank does not perform noticeably. There could be three reasons: the first reason is that the majority decoys on the train data (CASP10 dataset) are Best 150 decoys of CASP10, so the proposed method shows a better capability to rank the Best 150 decoys than to rank the Select20 decoys of CASP11. The second reason is that some target proteins on the CASP11 Select 20 dataset just have very low quality decoys, the proposed method could not distinguish them reasonably based on the model pairwise comparison. For instance, the mean decoy GDT_TS score of target T0776 is 61.33, the Pearson correlation coefficient between prediction scores and GDT_TS scores of this target is 0.97. But for the target T0808, the mean decoy GDT_TS score is 7.15 and the Pearson correlation coefficient is −0.06. So the proposed method will not perform well if all decoys have very low qualities, which needs to be promoted in our future study. The third reason is there are much fewer decoy models with high quality on the CASP11 select 20 dataset, so it is harder to obtain high quality decoy models as reference models. In order to verify this case, we selected 10 protein targets whose loss scores were very large (at least 22 on GDT_TS score) and extracted the first five decoy models from CASP11 best 150 dataset by using MQAPRank. Then, these five decoy models were taken as reference models of Quasi-MQAPRank to estimate decoy model qualities of the corresponding protein target on CASP11 select 20 dataset. Compared with the initial performance, the Quasi-MQAPRank method achieved a better performance with new reference models, details are show in Supplementary Table S3.

Supplementary Table S4 (GDT_TS score) and Table S5 (TM-score) show the performance of other methods on the CASP10&CASP11 dataset that have been assessed on the 3DRobot dataset. The corresponding ROC curves are provided in Supplementary Fig. S3 (GDT_TS score) and Fig. S4 (TM-score). Compared with the performance on the 3DRobot dataset, it’s clear that all of these methods have particularly poor performance on the CASP10&CASP11 dataset and different methods have different decrease levels. For the universal poor performance on the CASP10&CASP11 dataset, one most important reason could be that, for a specified target protein, the model quality distributions in the CASP10&CASP11 dataset are centralized, while the distributions in the 3DRobot dataset are more uniform (see Supplementary Fig. S6). This could be proved by the difference of the average standard deviation, which is calculated as the sum of the standard deviation of every target protein model quality distributions divided by the total number of target proteins. The average standard deviation reflects the diversity of data distribution, which will be larger if the data distribution were more uniform, and we get 21.51 (0.21 based on TM-score) for the 3DRobot dataset and 4.36 (0.04 based on TM-score) for the CASP10&CASP11 dataset based on the GDT_TS score. It clearly shows that the model quality distribution of the 3DRobot dataset is more uniform and the models will be easier to be distinguished because of the obvious gap of quality. However, the models’ qualities in the CASP10&CASP11 dataset are very close to each other and are hard to be distinguished by various methods. In CASP11, there are similar performances of various methods on the Best 150 dataset and Select 20 dataset. The Best 150 dataset is a dataset comprised of the best 150 models submitted on a target according to the benchmark consensus method and the model quality distributions is centralized, while the Select20 dataset is a dataset comprised of 20 models spanning the whole range of server model difficulty on each target and the model quality distributions is more uniform. As shown in Table 2, most of the methods have better performance on the Select 20 dataset.

We calculated the p-values in Student’s t-test as the statistical significance for the difference in PMCC scores between quasi single-model and single-model methods on CASP11 dataset. As shown in Supplementary Table S6 and Table S7, most of the p-values in Student’s t-test between the proposed methods and other methods are small sufficiently, demonstrating that the differences are statistically significant.

### Quasi single-model method achieves better performance than single-model method

In the last three CASPs^4^,^5^,^37^, the cluster-based methods generally outperform the single-model methods. The basic hypothesis of the clustering-based methods is that the conformations predicted more frequently are more likely to be correct compared with the structural patterns occurring in only a few models.

Here, we extend the single-model method by using the top five models as reference models. The predicted qualities of other models are calculated by the average similarity (GDT_TS score or TM-score) with these reference models. As shown in all tables and figures, the quasi single-model method can further improve the performance in comparison with the single-model method, and achieve better results in almost all evaluation metrics, which shows that the quasi single-model strategy has the capacity to improve the performance of the single model methods to some extent.

Furthermore, compared with the cluster-based methods, the quasi single-model methods are not sensitive to the distribution of decoy qualities and more capable of selecting the best model from model pool. The proposed quasi single-model method not only has good ability for selecting the best model but also achieves a comparative performance on Pearson correlation coefficient, which shows a good prospect of itself.

### The rank-based method outperforms the classification-based method and the regression-based method

The traditional strategy of protein structure prediction aims to find the model with the lowest potential, so the ideal prediction method could discriminate the native structure from other decoy models. In this study, a classification-based method is implemented and compared with the proposed learning-to-rank method. The classification-based method uses the native structures as the positive samples and all decoy models as negative samples. The same features are used for both methods. On the 3DRobot dataset, the classification-based method achieves a wmPMCC value of 0.30 and an AUC value of 0.68 based on GDT_TS score by using five-fold cross-validation, which are significantly lower than those achieved by learning-to-rank based method. The reason is that the classification-based method takes all the decoy models as the same. So the decoy models should be considered to develop an effective potential for protein structure prediction.

A regression-based method is also implemented based on SVMlight^40^ using the same features with learning-to-rank based method, and its output space is the GDT_TS score of corresponding decoy model. On the 3DRobot dataset, the regression-based method achieves a wmPMCC value of 0.88 and an AUC value of 0.94 by using five-fold cross-validation, which are higher than those achieved by classification-based method but still lower than those achieved by learning-to-rank based method.

Compared with the classification-based method and the regression-based method, evaluation details are provided in Supplementary Table S8, the learning-to-rank based method is more appropriate for evaluating the protein model quality.

### Performance differences using different structure similarity metrics

In order to evaluate the performances of various reference methods and our methods, the GDT_TS score (global distance test total score)^1^ and the TM-score^25^ are used as structure similarity metric at the same time.

The GDT_TS structure similarity metric will identify multiple maximum substructures associated with several different threshold cutoffs (1, 2, 4, and 8 Å), and is defined as the average coverage of the target sequence of the substructures with the four different distance thresholds.

The TM-score is defined as equation (4):


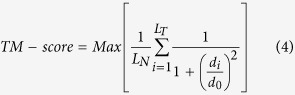


where *L*_*N*_ is the length of the native structure, *L*_*T*_ is the length of the aligned residues to the template structure, *d*_*i*_ is the distance between the *i*th pair of aligned residues and *d*_*0*_ is a scale to normalize the match difference.

On the 3DRobot dataset, there is no significant difference between the performance using the GDT_TS score and that using the TM-score. While on the CASP11 dataset, all of these methods have a better performance using the GDT_TS score, compared with the corresponding performances using the TM-score. Such phenomenon is reasonable since most of the methods participate in the CASP competition in which the GDT_TS score is the official metric for structure comparison.

## Materials and Methods

### Dataset

Three decoy model datasets (3DRobot dataset, CASP10 dataset, CASP11 dataset) are used in this study. Firstly, we used 3DRobot dataset to make a preliminary benchmark test by the five-fold cross-validation, and then, in order to achieve an objective evaluation and a fair comparison with other methods, we used the CASP10 dataset as the training set and the CASP11 dataset as the test set.

3DRobot dataset: The decoy models are downloaded from the website of 3DRobot decoys (http://zhanglab.ccmb.med.umich.edu/3DRobot/decoys/), which is generated by the 3DRobot^41^, a program devoted for automated generation of diverse and well-packed protein structure decoys. This decoy model dataset includes the structural decoys of 200 non-homologous proteins comprising by 48 α, 40 β, and 112 α/β single-domain proteins with length ranging from 80 residues to 250 residues. Each protein has 300 structural decoys with RMSD ranging from 0 Å to 12 Å. A benchmark assessment of our method was performed on this dataset by using the five-fold cross-validation.

CASP10 dataset: Referring to the model quality estimates in CASP10^4^, We downloaded all of the decoy models whose target protein structures have been published on the Protein Data Bank^42^ and removed the incomplete decoy models. Finally, we get 11501 decoy models of 91 target proteins, whose lengths range from 33 residues to 770 residues. This dataset is used as the final training dataset of our method.

CASP11 dataset: In a similar way with the CASP10 dataset, we make a data collection from the model quality assessment in CASP11^5^. Firstly, we downloaded all of the decoy models whose target protein structures have been published, and then, we removed the incomplete decoy models, finally, we get 8831 decoy models of 87 target proteins with length ranging from 44 residues to 525 residues. According to the design of CASP, these decoy models are divided into two datasets: the Best 150 dataset (7457 decoy models, a dataset comprised of the best 150 models submitted on a target according to the benchmark consensus method) and the Select 20 dataset (1682 decoy models, a dataset comprised of 20 models spanning the whole range of server model difficulty on each target). Using these two datasets, we could make a sufficient and fair evaluation on various kinds of model quality assessment (MQA) methods.

Supplementary Fig. S5 shows the entire quality score (GDT_TS and TM-Score) distributions of the decoy models in the 3DRobot dataset and CASP10&CASP11 dataset (merging CASP10 and CASP11 dataset together). The entire quality score distributions are uniform for both datasets, however, there exists an obvious difference between the two datasets when taking each target protein’s quality score distribution into account. For 3DRobot dataset, the decoy models of each target protein have uniform quality score distributions, but the distributions are very concentrative on the CASP10&CASP11 dataset, as shown in Supplementary Fig. S6. This distribution difference will result in a different performance for model quality assessment methods, we have analyzed the difference in the results and discussions section.

## Methods

### Feature extraction

Two kinds of features from the decoy model are extracted: well-established knowledge-based mean force potentials and the evaluation scores of other state-of-the-art programs for model quality assessment of proteins.

The knowledge-based potentials used in our method include Boltzmann-based potentials^43^, the DFIRE potential^44^, the DOPE potential^45^, the GOAP potential^46^ and the RWplus potential^47^.

The Boltzmann-based potentials are widely used mean force potentials that is derived from the inverse Boltzmann law, and the corresponding non-linear form are proposed in our previous study^43^. The five Boltzmann-based potentials include the DIH potential (a single-body residue-level Boltzmann-based potential^48^, which is obtained from the propensity of each amino acid for each dihedral Φ/Ψ class), the DFIRE-SCM potential (a two-body residue-level Boltzmann-based potential^49^, whose reference state is distance-scaled, finite ideal-gas reference state), FS potential (a two-body atom-level Boltzmann-based potential^50^), HRSC potential (a two-body residue-level linear potential, which is evaluated by the arithmetic sum of pairwise interactions corresponding to each amino acid combination at a particular contact distance^51^), T32S3 potential (a two-body atom-level linear potential with 32 type of atoms and 3 distance bins^52^).

The DFIRE potential is a distance-dependent structure-derived potential, which sums the interactions of all pairs of non-hydrogen atoms (167 atomic types).

The DOPE (Discrete Optimized Protein Energy) potential is based on an improved physical reference state that corresponds to non-interacting atoms in a homogeneous sphere with the radius dependent on a sample native structure. Its variants (DOPE-normal (Normalized DOPE by z score) and DOPE-HR (the bin size is 0.125 Å, a higher resolution than DOPE)) are also used.

The GOAP potential is a generalized orientation and distance-dependent all-atom statistical potential, which depends on the relative orientation of the planes associated with each heavy atom in interacting pairs.

The RWplus potential is based on the pair-wise distance-dependent atomic statistical potential function RW^53^, and contains a side-chain orientation-dependent energy term.

The evaluation scores from other model quality assessment programs are also extracted as additional features, which include the Frst^54^, ProQ^6^, RFMQA^11^, SIFT^55^ and SELECTpro^56^ software.

The Frst software’s output is based on four knowledge-based potentials: RAPDF potential, SOLV potential, HYDB potential, and TORS potential, and the Frst energy is a weighted linear combination of the four potentials. Besides the combination potential, the individual potentials are also used as the features in our method.

The ProQ is a neural-network-based method to predict the protein model quality. It uses structural information which contains the frequency of atom contacts and residue contacts, solvent accessibility surfaces, the fraction of similarity between predicted secondary structure and the secondary structure in the model, and the difference between the all-atom model and the aligned C-alpha coordinates from the template.

The RFMQA is a random forest based model quality assessment using structural features and knowledge-based potential energy terms. Here we used an analogous strategy as RFMQA to extract four protein secondary structure features and two solvent accessibility features. For protein secondary structure features, the focus is the consistency between predicted and actual secondary structures of a target protein. For each decoy model, we use DSSP^57^ to calculate its secondary structures and PSIPRED^58^ to predict the secondary structures of the target sequence. The fraction of consistent secondary structural element (alpha-helix, beta-strand and coil) between the DSSP label and the PSIPRED output is calculated by dividing the consistency number by its total chain length, and the total consistency RFMQA-SS-total score is also used as a feature. For solvent accessibility features, the absolute solvent accessibility of the model is computed by DSSP and relative solvent accessibility is computed by ACCpro5^59^. These two vectors are compared and transformed into a Pearson Correlation Coefficient and a cosine value as two features.

The SIFT is a program that uses averaged (i.e. amino acid independent) radial distribution functions (RDF) to discriminate properly packed models from misfolded ones. It produces two alternative scores: one based on RDF only and the other based on a combination of RDF and other sequence-independent filters.

The SELECTpro is a structure-based model assessment method derived from an energy function comprising physical, statistical, and predicted structural terms that include predicted secondary structure, predicted solvent accessibility, predicted contact map, β-strand pairing and side-chain hydrogen bonding.

### Learning to rank

In the area of machine learning, learning to rank is a method to construct a ranking strategy which can sort new objects according to their relevance or importance to the target object. Learning to rank has been applied effectively to solve information retrieval problems, such as document retrieval^30^, collaborative filtering^31^, Spam Detection^32^, etc. Specifically, according to the four pillars of machine learning, which are input space, output space, hypothesis and loss function, the existing learning-to-rank algorithms can be categorized into three approaches: pointwise approach, pairwise approach, and listwise approach, and different approaches model the process of learning to rank in different ways.

One of the advantages of pointwise and pairwise approaches is that existing methodologies on regression and classification can be directly applied to the learning task. Furthermore, pairwise approaches generally outperform pointwise approaches and have been successfully applied to various information retrieval applications. The pairwise approaches take pairs of documents (represented as feature vectors) as instances for learning, and formalize the task of learning to rank as that of classification. In learning, it first collects document pairs from the ranking lists, and then assigns a label representing the relative relevance or importance of the two documents for each pair. The final process is to train a regression or classification model with the labeled data and to make use of the model for ranking new data^30^.

For a list of decoy models of a certain protein target, we could consider model quality assessment problem as ranking problem. For a protein target, we describe the match between target *t* and model *d* using feature vector Φ(*t*, *d*) and get ranking functions as equation (5):


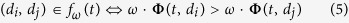


where *d*_*i*_ and *d*_*j*_ denote different decoy models, *f* is the ranking function, and ω is a weight vector that is adjusted by learning.

Like in classification SVM, we introduce slack variables and get the following optimization problem (equation (6)):

*minimize:*


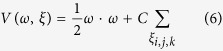


*subject to*:


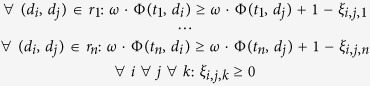


where *V* is the objective function, ξ is the slack variable, *C* is a parameter that allows trading-off margin size against training error, *r* represents the decoy model pair set of protein target and *k* is the subscript of constraints.

By rearranging the constraints, we get a new optimization problem (equation (7)) which is equivalent to that of SVM classification:


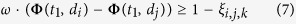


This problem can be solved using decomposition algorithms those used for SVM classification.

In this study, the pairwise ranking via-classification approach has been adopted to model quality assessment, and the SVM^rank^ program^60^ is used as the implement. The kernel function is taken as the linear kernel and the parameters are optimized with five-fold cross validation on the dataset.

## Conclusion

Evaluating the quality of protein models in perspective is one of the key stages of protein structure prediction and is an open problem. This study firstly presents a learning-to-rank framework named as MQAPRank which ranks the decoy models of a target protein by their relative qualities, and then uses the quasi single-model methodology to extend the method named as Quasi-MQAPRank. To evaluate the ability of the proposed methods for model quality assessment, the five-fold cross-validation is made on the 3DRobot dataset at first. The result shows that the proposed methods consistently outperform all other methods whose outputs are taken as features of the proposed method and that the quasi single-model method makes better performance. Reference to the CASP11 evaluation procedures, we train our method using CASP10 dataset and make tests on the CASP11 dataset. The proposed methods still perform well compared with other leading methods in corresponding categories. On the CASP11 Best 150 dataset, the Quasi-MQAPRank method not only outperforms other methods in the quasi single-model category in terms of all evaluation metrics, but also achieves a better performance even compared with the top established clustering-based methods, which indicates that the quasi single-model method has good prospects on big datasets. On the CASP11 Select 20 dataset, these two methods do not perform noticeably, which needs to be promoted in the future study. In general, the results show that the proposed methods provide the state-of-the-art performance and are available for genome-wide protein structure evaluation.

## Additional Information

**How to cite this article**: Jing, X. *et al*. Sorting protein decoys by machine-learning-to-rank. *Sci. Rep.* **6**, 31571; doi: 10.1038/srep31571 (2016).

## Supplementary Material

Supplementary Information

## Figures and Tables

**Table 1:** The comparative results of the proposed methods with other methods on 3DRobot dataset based on GDT_TS score.

Method	wmPMCC↑	PMCC↑	AUC↑	Loss↓	Top↑
**Quasi-MQAPRank(this study)**	**0.99**	**0.99**	**0.99**	**0.80**	140
**MQAPRank (this study)**	0.95	0.88	0.95	**0.80**	140
ModFOLDclust2	0.95	0.90	**0.99**	7.51	13
DFIRE	0.88	0.14	0.95	7.56	30
DOPE	0.89	0.66	0.95	4.45	72
GOAP	0.91	0.55	0.96	3.88	85
RWplus	0.87	0.13	0.95	7.20	32
Frst	0.86	0.78	0.94	3.11	109
ProQ	0.86	0.69	0.93	12.17	47
RFMQA	0.92	0.87	0.96	1.70	**143**
SIFT	0.63	0.55	0.79	15.31	32
SELECTpro	0.79	0.60	0.92	17.69	8
HRSC	0.60	0.15	0.81	18.38	6
Nonlinear-HRSC	0.81	0.56	0.91	11.07	14

The ModFOLDclust2 is a clustering method, other compared methods are listed in “feature extraction” section.

**Table 2:** The comparative results of the proposed methods with other thirteen methods from CASP11 on CASP11 dataset based on GDT_TS score.

Category	Method	Best 150					Select 20				
		wmPMCC	PMCC	AUC	Loss	Top	wmPMCC	PMCC	AUC	Loss	Top
clustering-based	Pcons-net	**0.71**	**0.94**	**0.98**	5.28	3	0.91	0.93	**0.98**	**2.79**	**57**
	Wallner	0.70	**0.94**	**0.98**	**4.87**	**4**	0.86	0.94	**0.98**	5.32	53
	DAVIS-QAconsensus	0.68	**0.94**	**0.98**	7.74	0	0.90	**0.95**	**0.98**	5.51	48
	MULTICOM-REFINE	0.68	**0.94**	**0.98**	7.62	0	0.90	0.92	**0.98**	5.20	50
	ModFOLDclust2	0.66	**0.94**	**0.98**	7.28	0	0.86	**0.95**	**0.98**	5.36	47
	MQAPmulti	0.59	0.81	0.93	9.06	0	**0.93**	0.91	0.97	5.14	44
quasi single-model	**Quasi-MQAPRank (this study)**	**0.74**	**0.95**	**0.98**	**4.84**	**5**	0.77	0.91	0.97	8.29	37
	MQAPsingleA	0.65	0.75	0.90	8.95	1	**0.92**	0.88	0.95	3.64	52
	MQAPsingle	0.56	0.75	0.90	9.51	3	0.89	0.86	0.94	6.34	41
	ModFOLD5_single	0.53	0.92	0.96	10.31	0	0.91	**0.96**	**0.99**	3.65	**53**
	nns	0.54	0.89	0.95	7.75	**4**	0.83	0.91	0.97	**3.22**	52
	ConsMQAPsingle	0.53	0.73	0.89	8.37	1	0.87	0.82	0.94	5.18	47
single-model	**MQAPRank (this study)**	**0.51**	0.75	0.90	**4.84**	5	0.64	0.65	0.77	8.29	37
	MULTICOM-CLUSTER	0.43	**0.79**	**0.91**	7.06	7	**0.71**	**0.82**	0.92	9.47	34
	VoroMQA	0.43	0.55	0.80	7.31	7	0.60	0.61	0.83	10.76	31
	MULTICOM-NOVEL	0.41	0.69	0.89	6.89	**8**	0.69	0.73	0.91	9.08	37
	ProQ2	0.38	0.76	**0.91**	*6.34*	5	0.70	0.79	**0.93**	**8.14**	**40**
