# Supplementary material for: Sorting protein decoys by machine-learning-to-rank
Source: Sci Rep. 2016 Aug 17;6:31571. doi: 10.1038/srep31571 (PMC4987638; doi:10.1038/srep31571)
Supplement: Supplementary Information [file srep31571-s1.pdf]

Sorting protein decoys by machine-learning-to-rank

Xiaoyang Jing, Kai Wang, Ruqian Lu and Qiwen Dong

Supplementary Information

Table S1. The comparative results of the proposed methods with other methods on 3DRobot dataset based on TM-score.

Method	wmPMCC↑	PMCC↑	AUC↑	Loss↓	Top_1↑
**Quasi-MQAPRank(this study)**	**0.99**	**0.99**	**0.99**	0.0131	**149**
**MQAPRank (this study)**	0.94	0.88	0.93	**0.0057**	**149**
ModFOLDclust2	0.97	0.87	0.99	0.0619	13
DFIRE	0.88	0.33	0.93	0.0657	30
DOPE	0.89	0.63	0.94	0.0393	73
GOAP	0.91	0.67	0.95	0.0337	85
RWplus	0.87	0.32	0.93	0.0625	32
Frst	0.86	0.74	0.91	0.0240	109
ProQ	0.86	0.72	0.92	0.0995	47
RFMQA	0.92	0.87	0.95	0.0145	142
SIFT	0.63	0.49	0.78	0.1308	32
SELECTpro	0.79	0.48	0.91	0.1455	8
HRSC	0.60	0.34	0.80	0.1504	6
Nonlinear-HRSC	0.81	0.63	0.90	0.0922	12

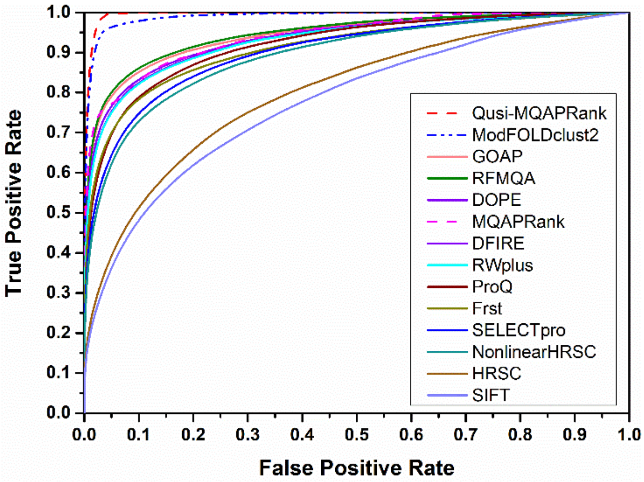

Figure S1. The ROC curves of compared methods on the 3DRobot dataset based on TM-score.

Table S2. The comparative results of the proposed methods with other fifteen methods taking part in the CASP11 on CASP11 dataset based on TM-score.

Category	Method	Best 150					Select 20				
		wmPMCC	PMCC	AUC	Loss	Top	wmPMCC	PMCC	AUC	Loss	Top
**clustering-based**	Pcons-net	**0.56**	**0.81**	**0.94**	0.07	1	0.84	**0.90**	**0.98**	**0.03**	**30**
	Wallner	**0.56**	**0.81**	**0.94**	**0.06**	**2**	0.80	0.89	**0.98**	0.05	29
	DAVIS-QAconsensus	0.52	**0.81**	**0.94**	0.08	0	0.86	0.87	**0.98**	0.05	26
	MULTICOM-REFINE	0.52	**0.81**	**0.94**	0.08	0	0.86	0.86	0.97	0.05	26
	ModFOLDclust2	0.53	**0.81**	**0.94**	0.07	0	0.85	0.88	0.97	0.04	27
	MQAPmulti	0.46	0.61	0.86	0.08	1	**0.88**	0.86	0.97	0.04	26
**quasi single-model**	**Quasi-MQAPRank (this study)**	**0.68**	0.79	0.93	**0.04**	**10**	0.82	0.88	0.98	0.09	14
	MQAPsingleA	0.50	0.57	0.82	0.07	0	**0.88**	0.81	0.95	**0.03**	**31**
	MQAPsingle	0.41	0.56	0.82	0.09	0	0.82	0.78	0.94	0.06	24
	ModFOLD5_single	0.39	0.79	**0.94**	0.10	1	0.85	**0.90**	**0.99**	0.04	29
	nns	0.44	**0.80**	**0.94**	0.07	1	0.78	0.89	0.97	**0.03**	30
	ConsMQAPsingle	0.43	0.54	0.81	0.08	0	0.84	0.74	0.93	0.05	27
**single-model**	**MQAPRank (this study)**	**0.43**	0.60	0.83	**0.04**	**10**	0.64	0.66	0.89	0.09	14
	MULTICOM-CLUSTER	0.29	**0.70**	0.87	0.07	2	0.62	**0.77**	0.92	**0.08**	**17**
	VoroMQA	0.28	0.52	0.78	0.08	2	0.52	0.59	0.83	0.09	15
	MULTICOM-NOVEL	0.27	0.69	**0.88**	0.08	1	**0.65**	0.75	**0.93**	0.09	17
	ProQ2	0.30	**0.70**	0.87	0.06	3	**0.65**	**0.77**	0.92	**0.08**	**17**

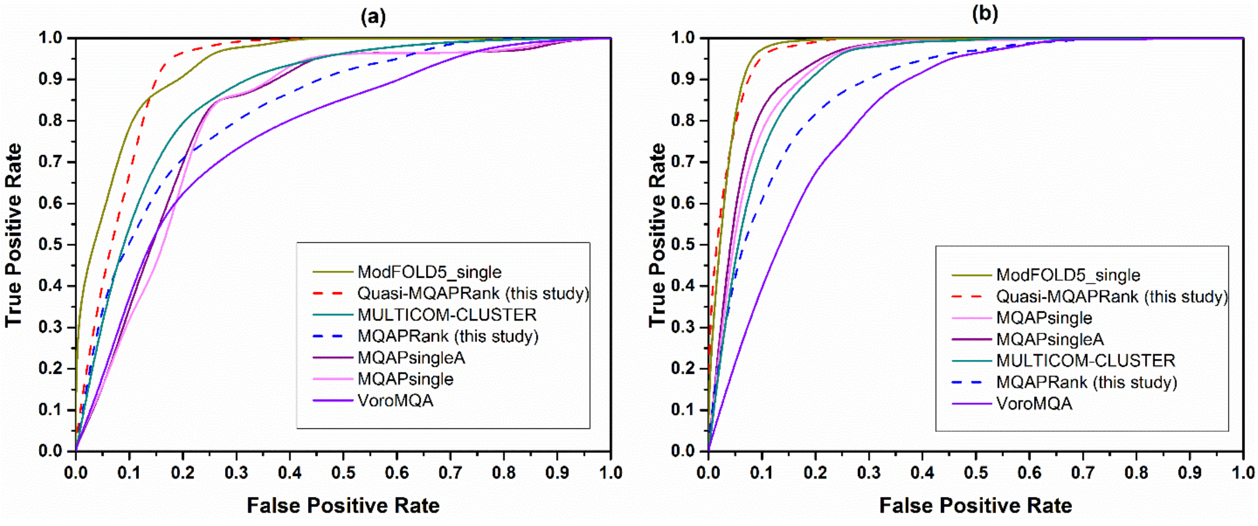

Figure S2. The ROC curves of compared methods on the CASP11 dataset based on TM-score. (a) The ROC curves for Best150 dataset and (b) the corresponding curves for Select20 dataset.

Table S3. The comparative results of the Quasi-MQAPRank method between initial reference models and new reference models on 10 CASP11 Select 20 targets based on GDT_TS score. The 10 targets are: T0833, T0786, T0764, T0768, T0819, T0800, T0815, T0780, T0857, and T0782.

Dataset	wmPMCC↑	PMCC↑	AUC↑	Loss↓	Top↑
**Initial reference models**	0.71	0.90	0.98	27.50	0
**New reference models**	**0.99**	**0.99**	**0.99**	**2.48**	**9**

Table S4. The comparative results of other methods on CASP10&CASP11 dataset based on GDT_TS score.

Method	wmPMCC↑	PMCC↑	AUC↑	Loss↓	Top_1↑
**DFIRE**	0.29	0.02	0.53	7.24	3
**DOPE**	0.29	0.48	0.75	5.88	5
**GOAP**	0.43	0.29	0.68	5.38	7
**RWplus**	0.22	0.01	0.52	7.21	3
**Frst**	0.29	0.42	0.72	6.62	9
**ProQ**	0.33	0.47	0.78	7.74	5
**RFMQA**	0.35	0.68	0.87	7.34	4
**SIFT**	0.25	0.23	0.66	7.73	5
**SELECTpro**	0.18	0.40	0.71	7.46	6
**HRSC**	0.04	0.03	0.51	8.93	0
**Nonlinear-HRSC**	0.13	0.29	0.70	8.80	5

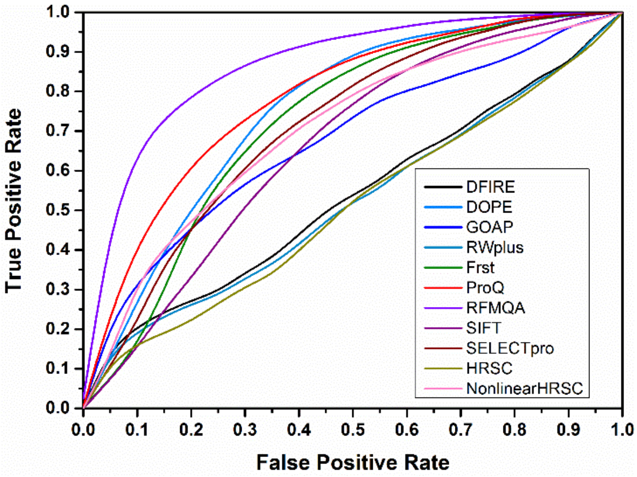

Figure S3. The ROC curves of compared methods on the CASP10&CASP11 dataset based on GDT_TS score.

Table S5. The comparative results of other methods on CASP10&CASP11 dataset based on TM-score.

Method	wmPMCC↑	PMCC↑	AUC↑	Loss↓	Top_1↑
**DFIRE**	0.18	0.35	0.69	0.0797	4
**DOPE**	0.25	0.25	0.61	0.0644	6
**GOAP**	0.26	0.49	0.77	0.0623	3
**RWplus**	0.14	0.33	0.68	0.0778	4
**Frst**	0.20	0.18	0.58	0.0764	5
**ProQ**	0.31	0.35	0.69	0.0705	4
**RFMQA**	0.36	0.41	0.70	0.0604	10
**SIFT**	0.13	0.05	0.58	0.0762	4
**SELECTpro**	0.24	0.12	0.51	0.0616	13
**HRSC**	0.01	0.29	0.67	0.0845	2
**Nonlinear-HRSC**	0.29	0.40	0.72	0.0803	6

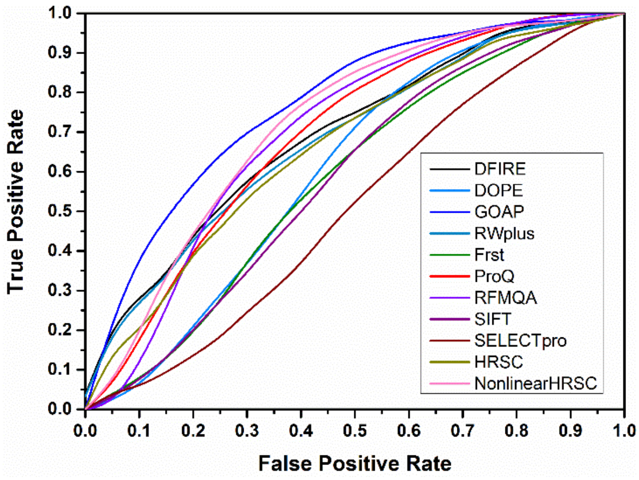

Figure S4. The ROC curves of compared methods on the CASP10&CASP11 dataset based on TM-score.

Table S6. The p-values in Student's t-test for the difference in PMCC scores between quasi single-model methods on CASP11 dataset

Methods	Quasi-MQAPRank	MQAPsingleA	MQAPsingle	ModFOLD5_single	nns	ConsMQAPsingle
Quasi-MQAPRank	1.0000	2.13E-05	0.0424	0.9442	0.0049	0.0382
MQAPsingleA	2.13E-05	1.0000	0.0633	0.0002	0.1050	0.0610
MQAPsingle	0.0424	0.0633	1.0000	0.0299	0.6195	0.8840
ModFOLD5_single	0.9442	0.0002	0.0299	1.0000	0.0220	0.0161
nns	0.0049	0.1050	0.6195	0.0220	1.0000	0.7028
ConsMQAPsingle	0.0382	0.0610	0.8840	0.0161	0.7028	1.0000

Table S7. The p-values in Student's t-test for the difference in PMCC scores between single-model methods on CASP11 dataset

Methods	MQAPRank	MULTICOM-CLUSTER	VoroMQA	MULTICOM-NOVEL	ProQ2
MQAPRank	1.00E+00	2.69E-07	2.14E-07	9.77E-08	1.38E-09
MULTICOM-CLUSTER	2.69E-07	1.00E+00	8.05E-01	6.41E-01	2.15E-01
VoroMQA	2.14E-07	8.05E-01	1.00E+00	4.72E-01	1.17E-01
MULTICOM-NOVEL	9.77E-08	6.41E-01	4.72E-01	1.00E+00	4.97E-01
ProQ2	1.38E-09	2.15E-01	1.17E-01	4.97E-01	1.00E+00

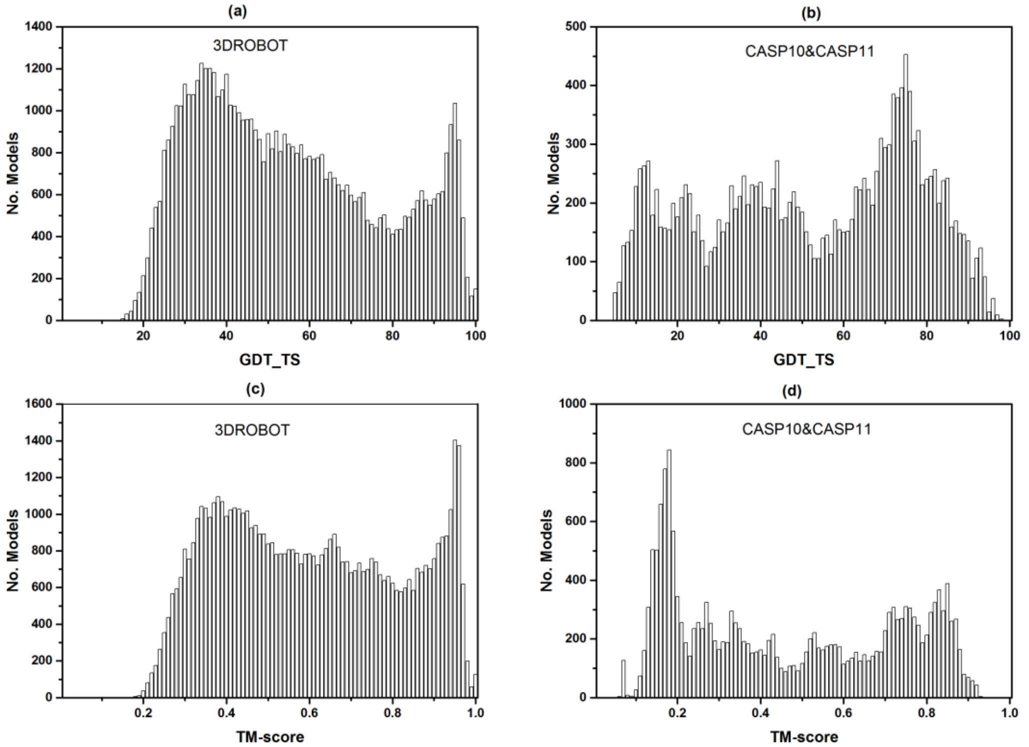

Figure S5. The quality score distributions of all decoy models. The GDT_TS score distributions in the 3DRobot dataset and CASP10&CASP11 dataset are shown in subfigure (a) and (b). The TM-Score distributions in the 3DRobot dataset and CASP10&CASP11 dataset are shown in subfigure (c) and (d).

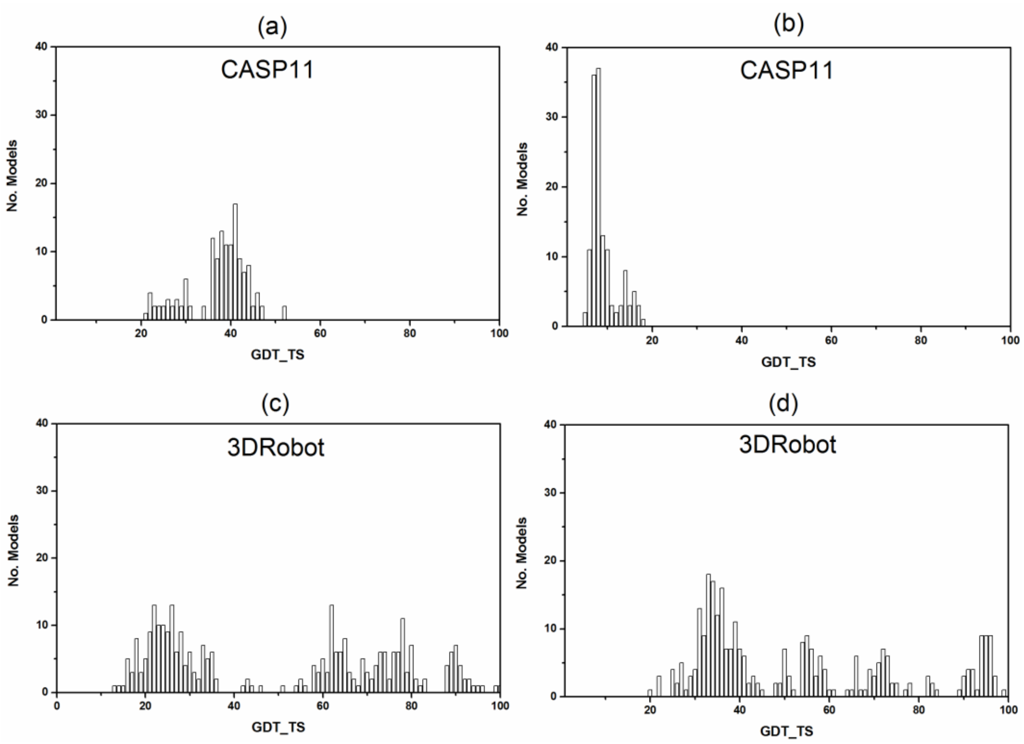

Figure S6. The quality score distributions of decoy models of four target proteins. The distributions of two target proteins ((a) T0803 and (b) T0781) in the CASP11 dataset are centralized, while the distributions of two target proteins ((c) 4IZXA and (d) 2WDSA) in the 3DRobot dataset are uniform.

Table S8. The comparative results of the proposed methods with the classification-based method and the regression-based method on 3DRobot dataset based on GDT_TS score

Method	wmPMCC↑	PMCC↑	AUC↑	Loss↓	Top_1↑
**Quasi-MQAPRank(this study)**	**0.99**	**0.99**	**0.99**	**0.80**	140
**MQAPRank (this study)**	0.95	0.88	0.95	**0.80**	140
**classification-based**	0.30	0.22	0.68	1.21	**156**
**regression-based**	0.88	0.62	0.94	3.79	101
